# In-Office Hyaluronic Acid Injection of Vocal Folds in Patients with Presbyphonia

**DOI:** 10.3390/jcm14030960

**Published:** 2025-02-03

**Authors:** Anastasiya Avdiyuk, Patricia Garnica, Ramón González-Herranz, Estefanía Miranda, Cristina García-García, Guillermo Plaza

**Affiliations:** 1 Otorhinolaryngology Department, Hospital Universitario de Fuenlabrada, C.P. 28042 Madrid, Spain; estefania-miranda@hotmail.com (E.M.); crisgar223@gmail.com (C.G.-G.); 2 Departamento de Especialidades Médicas y Salud Pública, Facultad de Ciencias de la Salud, Universidad Rey Juan Carlos, C.P. 28933 Madrid, Spain; p.garnica.2018@alumnos.urjc.es; 3 Otorhinolaryngology Department, Hospital Universitario Sanitas La Zarzuela, C.P. 28023 Madrid, Spain; rgherranz@salud.madrid.org

**Keywords:** hyaluronic acid, laryngoplasty, presbyphonia, vocal fold atrophy

## Abstract

**Objective**: to evaluate the advantages and disadvantages of injection laryngoplasty with hyaluronic acid in patients older than 65 years with presbyphonia. **Study Design**: a retrospective observational study. Setting: an academic secondary medical center. **Methods**: This study was performed using a group of patients diagnosed with presbyphonia who were treated using injection laryngoplasty with hyaluronic acid and underwent a minimum follow-up of 12 months. Subjective parameters such as the Voice Handicap Index–10 (VHI-10) and the GRBAS scale (grade, roughness, breathiness, asthenia, strain) were measured, as well as objective parameters such as the closure defect area. The medical records of patients undergoing this procedure during the 2020–2023 period were reviewed. An analysis of the demographic and clinical variables of the group was performed, as well as the values of the VHI-10, GRBAS, difference, and improvement of the area of closure defect before and after the procedure, along with the treatment duration and sensation of improvement. **Results**: The mean pre- and postoperative VHI-10 decreased from 26.8 to 19.6, showing significant differences (*p* = 0.007). The postoperative GRBAS mean score was 5.6 and normality can be assumed when it is below 9. Out of the 16 patients, 11 of them reported subjective improvement in their symptoms. More than half of them showed an improvement in the closure defect greater than 80%, with a significant reduction in the area (*p* < 0.001). **Conclusions**: hyaluronic acid injection in patients with presbyphonia produced a clear subjective improvement in voice quality and a decrease in the closure defect area.

## 1. Introduction

In recent years there has been a demographic transition, with a considerable increase in the population of people over 65 years old. This leads to the appearance of a series of degenerative changes that must be treated in order to increase quality of life [1]. One of them is the senile voice or presbyphonia, a dysfunction that appears from the age of 65, as a result of vocal fold aging or presbylarynx [2,3]. The impairment of communication that is generated by this condition can lead to social isolation [4].

Presbyphonia is the result of several changes that occur in the pharynx, larynx, and diaphragm, as well as muscle atrophy and sarcopenia [4]. In regard to the larynx, changes occur in the connective tissue, leading to a reduction in the content of hyaluronic acid (HA) and elastin, and an increase in collagen deposits. The vocal folds become less elastic and flexible, which causes a decrease in the vibrating wave. In addition, generalized muscular atrophy takes place, manifesting as vocal fold bowing. Some of the symptoms presented by patients include vocal fatigue, a hoarse and shortened voice, tone changes, difficulty in singing and speaking, and even aspiration [5,6,7]. Establishing a correct diagnosis and treating this dysfunction is important as it enhances the quality of life of these patients, who are otherwise not able to communicate normally in their environment [1].

Once vocal therapy has failed, there are some surgical strategies available to manage these patients. The least invasive surgical option consists of injection laryngoplasty using various materials such as HA or autologous fat. The aim of this technique is to introduce a substance that acts as a filler medializing the vocal folds, thereby reducing the closure defect, with minimal inflammatory effect and low risk of infection [1]. The decision behind the type of material used lies in the treatment duration. Those classified as short- to medium-term can be useful for evaluating the improvement of the closure defect with this technique and selecting a more durable material. A study conducted by Kharidia et al. showed that HA-based products were the preferred choice of injectable material used to perform this procedure of most laryngologists [8].

Furthermore, in recent years, the number of procedures performed in an office-based setting with awake and non-sedated patients has increased due to lower healthcare costs, less complications, and similar effectiveness compared to surgical interventions in an operating room [4,5].

In addition, older adults often have concomitant conditions that make them reluctant to undergo procedures that require going to the operating room, such as thyroplasty or fat infiltration. Therefore, in-office infiltration of HA is a good option [7].

This study aimed to evaluate the evolution of patients after office-based injection laryngoplasty with HA, assessing the possible advantages and disadvantages of the procedure. To do this, we considered the improvement of the closure defect area after infiltration, postoperative VHI-10, and the treatment duration.

## 2. Materials and Methods

This is a retrospective observational study of patients with presbyphonia treated with HA infiltration during the 2020–2023 period at a University Hospital, with a minimum follow-up of 12 months. This study was approved by our local Ethics Committee (23/99, 23 January 2024). In addition, the necessary annexes were submitted to the committee to ensure the protection of the data of the patients involved, as well as the researchers’ confidentiality commitment.

**Figure 1:**
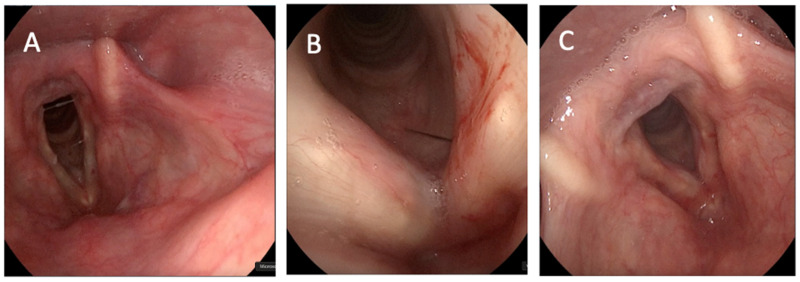
Cricothyroid injection laryngoplasty approach. (**A**) Pre-operative result. (**B**) Procedure. (**C**) Immediate result. Video on trans-cricothyroid HA infiltration can be found as Video S1 as supplementary material.

**Figure 2:**
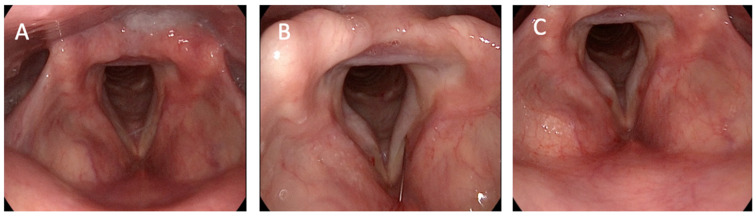
Thyrohyoid injection laryngoplasty approach. (**A**) Pre-operative result. (**B**) Procedure. (**C**) Immediate result. Video on trans-thyrohyoid HA infiltration can be found as Video S2 as supplementary material.

The inclusion criteria for this research were an age older than 65 years with diagnosis and follow-up of presbyphonia in our Voice Unit. All patients that underwent this procedure had previously completed at least 15 sessions of voice therapy. Patients underwent office-based injection laryngoplasty with HA, which was performed either through the trans-cricothyroid approach (Figure 1) or through the trans-thyrohyoid approach (Figure 2).

All patients were monitored for at least 12 months to assess treatment evolution, beginning with an initial video consultation and with another used for follow-up after the infiltration. A total of 18 patients were enrolled. This number can be explained by the fact that the study began in 2020, a time when the healthcare world was hit by the COVID-19 pandemic, which led many patients with other conditions to decide not to go to hospitals.

All patients were classified according to demographic (age, sex) and clinical variables (tobacco use, inhaler use) in order to determine possible risk factors. The parameter used to evaluate the quality of life of the patients was Voice Handicap Index Score (VHI). This is a widely used tool specifically designed to assess the quality of life of patients with voice problems. The extended version consists of 30 questions (VHI-30), while the shortened version consists of 10 questions (VHI-10). It assesses the impact on the physical, functional, and emotional spheres. The shortened version was used in this study due to its ease of comprehension. The pre- and postoperative VHI-10 scores were collected in order to examine any possible improvement in the patient’s quality of life after undergoing hyaluronic acid injection laryngoplasty. Some other parameters that were collected with the aim of assessing the results of this technique were the postoperative GRBAS score, closure defect area, difference and improvement of the closure defect after infiltration, treatment duration, and sensation of improvement. Unfortunately, pre-operative GRABS scores were not collected as they did not appear in all of the medical records reviewed.

The procedure was performed by the same ENT specialist under local anesthesia with 2% intratracheal lidocaine using a percutaneous approach via the trans-cricothyroid or trans-thyrohyoid approach. The middle third of the muscular space of the vocal folds was infiltrated with Restylane Lyft^TM^, thereby avoiding the Reinke space.

Once the patients to be included in the study were selected, we extracted the laryngeal stroboscopy recordings made with a flexible fibrolaryngoscope (11101 HD, Karl Storz, Tuttlingen, Germany) in the medical office. All stroboscopic videos were analyzed by specific experts and confirmed to have a closure gap at the start and to not have vocal fold paresis. At this point, the sample was reduced to sixteen patients, as one of them was lost to follow-up in the Voice Unit and, therefore, did not have a post-infiltration recording, and another one was excluded due to the impossibility of measuring his closure defect area due to artifacts in his recordings.

Finally, using the computer program ImageJ, version 1.54f, 2023 (NIH, Bethesda, MD, USA), we were able to obtain a relative measurement of the closure defect area from the fragments extracted from the stroboscopy recordings. To measure the defect area in a static image obtained from a stroboscopy, we first selected a video segment containing a complete and representative vocal cycle. From this segment, we extracted the frames corresponding to the full vocal cycle and identified the frame in which the vocal folds exhibit the smallest closure defect, representing the point of maximum approximation.

Using the selected frame, we employed the ImageJ software to perform the measurements. Initially, we measured the distance between the anterior commissure and the vocal process of the vocal fold, assigning this segment a relative value of 1000 units. This served as a reference scale for proportional measurements. Once the scale was established, we delineated the area corresponding to the closure defect by delimiting it using dots, and measured it in the same relative units. This process enabled precise quantification of the defect area in the selected image. The measurement of the closure defect area was performed this way, proving to be reproducible with the same image frame in 95% of all cases.

Qualitative variables were analyzed using the chi-square test, with a 95% confidence interval. For quantitative variables, as the sample included less than 50 patients, normality was evaluated using the Shapiro–Wilk test. Non-parametric Wilcoxon and Kruskal–Wallis tests were carried out as well. For this purpose, the SPSS Statistics program, version 27.0, was used.

## 3. Results

During the time period of this study, a total of 18 patients were treated in-office at our institution with injection laryngoplasty of HA. None of them developed any complications. All of the patients who underwent the procedure tolerated it well and postoperative pain was managed using first step pain medication, mainly acetaminophen. There were no patients that required hospitalization for pain management or ambulatory treatment with stronger pain medication, such as opioids. There were 12 women and 6 men. After applying the exclusion criteria, the sample size was reduced to 16 patients. The mean age was 69.63 (±5.97) years. The minimum follow-up time was 12 months and the maximum was 38. Regarding tobacco, there was a 1:1 ratio between smokers and non-smokers, whereas in the case of inhaler use, 6 out of 16 patients used them. To study the relation between smoking, as well as inhaler use, and the improvement after injection laryngoplasty, a non-parametric chi-square test was performed, which did not find any significant differences, as the *p* value was less than 0.05.

The results of the main variables studied are shown in Table 1.

VHI-10 scores were collected 1 month prior to the intervention as well as 3 months after it.

**Figure 3:**
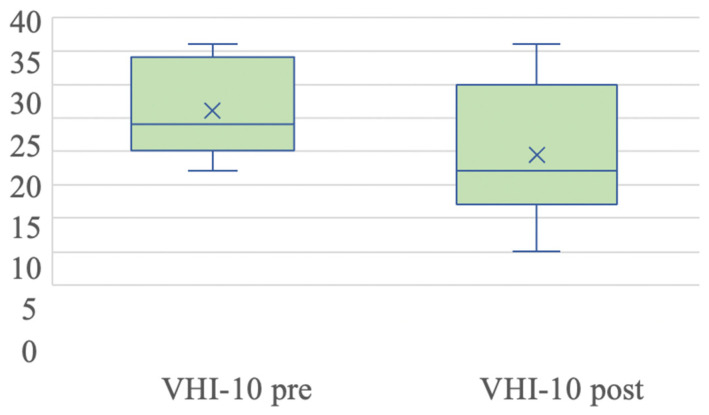
A boxplot of the evolution of pre- and postoperative VHI-10 scores. The values are presented as the mean (×), median (line), interquartile range (box), and 95% CI (error bars).

The pre- and postoperative mean score of VHI-10 ranged from 26.8 (±6.91) to 19.6 (±9.49) points (with a reduction due to treatment of 6.8 points) (Figure 3). Due to the baseline situation of one of the patients, we were unable to complete the questionnaire, omitting the VHI-10 in that case.

We only considered GRBAS values after treatment. However, taking into account that the mean score obtained was 5.6 (±2.34), normality was assumed since it was below 9. In addition, out of the 16 patients, 11 reported a subjective improvement in symptoms.

Of the 16 patients for whom the closure defect area could be calculated through the stroboscopy recordings, all of them showed an improvement in the defect area, with the mean being 18,873.93 (±16,286.92). In 11 of them, the improvement was greater than 80% after infiltration, and in 5 of them, despite it being less than 80%, the improvement was also quite big as it was greater than 50%. In addition, we also analyzed whether patients who presented a greater improvement in the defect closure area experienced a longer treatment duration as well. A duration of 9 months was considered as the reference since there are several studies that suggest a treatment duration up to this number. Taking into account this treatment duration, the results were very similar in both groups of patients. In the case of VHI-10 scores, taking 19 as the reference value, the results in both groups were also very similar (Table 2).

With a confidence interval of 95%, the chi-square value obtained in both cases was *p* > 0.05, so the results were not statistically significant. This means that the improvement of the closure defect due to infiltration is not clearly associated with the duration in months of such improvement or with the decrease in VHI-10 observed after treatment.

For the study of the VHI-10 scores, a non-parametric Wilcoxon test was performed, finding significant differences (*p* = 0.007) between the pre- and postoperative infiltration results.

**Figure 4:**
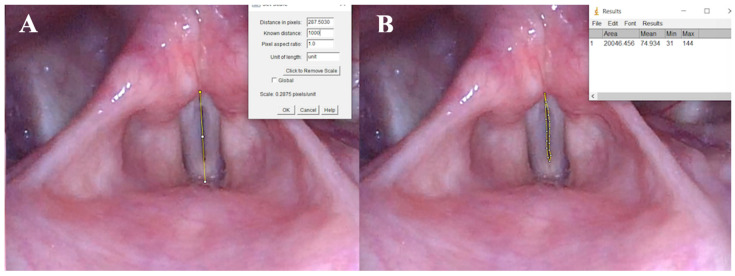
Measurement of glottic area using ImageJ program. (**A**) Calibration. (**B**) Delineation of glottic area. Video on defect area measurement using the ImageJ program can be found as Video S3 as supplementary material.

In the case of the closure defect area, another non-parametric Wilcoxon test was performed, finding a significant reduction in the described area thanks to IL with HA (*p* < 0.001) (Figure 4).

Finally, the previously described variables, the VHI-10 score and closure defect area, were analyzed together both before and after treatment using a non-parametric Kruskal–Wallis test. In this case, a higher level of statistical significance was obtained in the closure defect area (*p* < 0.001) than in the VHI-10 score (*p* = 0.029), both of which were very satisfactory.

## 4. Discussion

Currently, there are several techniques that have been developed for the treatment of presbyphonia, with the treatment of choice being a combination of vocal therapy and augmentation procedures [3,7]. As for injection laryngoplasty, in addition to the use of HA analyzed in this retrospective study, substances such as calcium hydroxyapatite, autologous fat, and platelet-rich plasma are available for this purpose as well [1,9]. A study conducted at the Institute of Physiology and Pathology of Hearing in Warsaw showed that injection laryngoplasty is a safe and effective procedure for treating glottic insufficiency in elderly people with presbyphonia [10]. A study carried out at the same institution in 2016, this time analyzing the acoustic characteristics of voice in patients that underwent this procedure, demonstrated a significant improvement in the quality of voice [11].

A systematic review of this technique carried out by Bové et al. showed that there has been a significant increase in its use in hospitals due to the advancement of new technologies and materials, as well its status as a cost-effective treatment option [12]. There are clear advantages when performing these procedures in medical offices without the need to use general anesthesia, such as being able to receive direct feedback regarding the glottic closure and voice quality during the injection. In addition, it allows clinicians to treat older patients who have contraindications preventing them from undergoing surgery or who want to avoid it. Patients selected for this technique must be cooperative and have minimal gag reflex [13].

In addition, the rate of complications associated with this technique has been reported to be very low. Some of these include edema, the formation of foreign body granuloma, ulceration, and hypersensitivity reactions, among others [14]. However, the rate of hypersensitivity reactions has experienced a decrease since the year 2000, associated with a decrease in the protein load in the materials used [15,16]. In a study conducted by Dominguez et al., the rate of inflammatory complications was 3.8% [15], while Enver et al. reported a total complication rate of 1.9%, which is very low as well [14].

The observed improvement in the closure defect, as well as the good medium-term duration, together with the degree of satisfaction shown in most patients, makes it possible to consider injection laryngoplasty with HA as a previous step to other more durable and more effective treatment options. According to a study by Molteni et al., the success of HA lies in the fact that it is widely expressed in the lamina propria of the vocal folds, and it is believed to intervene in the formation of the mucosal wave [17]. There are different types of HA that can be used, as shown in a systematic review performed by Švejdová et al. Juvederm and Restylane—large-particle HA and low-particle HA— showed the lowest mean VHI-30 score after 4 to 6 weeks of injection [18]. The approximate duration of this material is between four and six months, although several studies suggest a period of up to nine months. Regarding treatment duration, in our study, where injections with Restylane were used, a duration of voice improvement superior to nine months was reported in eight patients. Pei et al. suggest that a long-term effect occurs through a better psychosocial quality of life due to early involvement in social participation [19]. These data are consistent with those observed in the study conducted by Reiter and Bosch, in which a duration of voice improvement lasting up to twelve months was reported in eleven out of nineteen patients using this same material. These authors also observed that Restylane’s efficacy might be comparable to that of collagen and autologous fat [20].

It is important to remember that the benefit of injection laryngoplasty using hyaluronic acid is not limited solely to the improvement in voice quality, but also to the improvement of dysphagia caused by the pre-existing gap between the vocal cords, as reported by authors such as Alaskarov et al. [21].

Despite these results, the efficacy of injection laryngoplasty is greater in patients with vocal fold paralysis in comparison to vocal atrophy, as shown in reports such as that of Carroll et al. [22]. In addition, surgical success in patients with vocal fold atrophy is more limited as well. Thus, the treatment must be chosen very carefully [23].

Some of the surgical procedures that have proven to be more lasting or even permanent are autologous fat injection laryngoplasty or medialization thyroplasty. As described by Kumai et al., biocompatibility, as it is an autologous tissue, and the amount of stem cells may be responsible for the long-term effect of fat injection [24]. Studies like the one conducted by Shaw et al. reported histological evidence of viable fat 18 months after injection [25]. These conclusions are also supported by a study presented in 2022, in which follow-up was carried out on a sample of 18 patients with presbyphonia treated with autologous fat infiltration. It was shown that fat infiltration can be considered as a potential permanent option for all patients [26]. Several studies, like the one conducted by Zelenìk et al., showed subjective improvement in voice quality 1 year after the procedure in 64.3% of patients that underwent this procedure [27]. This percentage may reach 83%, as shown by Benninger et al. [28]. Nevertheless, long-term voice results using fat injection laryngoplasty are limited as the rate and amount of reabsorption are difficult to predict [29,30].

Although the results of injection laryngoplasty are quite good, the role of vocal therapy should not be overlooked. Authors such as Jeong et al. suggest its beneficial role when patients undergo it even after the procedure, as it helps maintain an improved voice quality for more than 6 months after the injection [31].

Medialization, or type 1 thyroplasty, is a procedure based on the placement of an implant between the thyroid cartilage and the vocal muscle in order to medialize the vocal folds. Dominguez et al. conducted a study comparing autologous fat injection with medialization laryngoplasty in patients with vocal fold atrophy. The results obtained were that both have short-term voice improvement, but the long-term effect of fat in most cases is limited. However, the surgical group showed a significant improvement in VHI-10 and glottal function index in the long term [32].

Haddad et al. [33] recently reviewed the outcomes of medialization procedures and found that the overall subjective improvement was greater in cases of vocal fold paralysis compared to age-related voice changes. This difference is likely due to the gradual onset and milder dysphonia experienced by elderly patients, making them less perceptive to postoperative changes. Although medialization enhances vocal fatigue and increases vocal volume, issues such as roughness or hoarseness tend to persist in presbyphonia. This persistence occurs because, despite achieving glottal closure, the stiffness of the vocal folds remains unaltered [34].

A study conducted by Sachs et al. [5] comparing injection laryngoplasty and thyroplasty in elderly patients showed no significant differences in normalized glottal gap area, true vocal fold width, or various acoustic and aerodynamic parameters. However, subjective assessments indicated superior results for thyroplasty.

It is important to recognize that medialization procedures are not without risk. Hyaluronic acid injections can, in rare cases, provoke an inflammatory response, which may range from localized vocal fold edema to more severe airway edema accompanied by dyspnea and stridor. Such reactions usually manifest within 24 to 48 h following the procedure and may result from allergic reactions, ischemia, vascular occlusion, or infection due to injection device contamination [14,35].

According to Haddad et al. [33], surgical intervention should be considered for patients with significant vocal demands, large glottal gaps unresponsive to voice therapy, or difficulty complying with therapy sessions. Both injection laryngoplasty with hyaluronic acid and medialization thyroplasty are viable options, with the former offering at least one year of effectiveness through injections and the latter providing a more permanent solution [5].

**Figure 5:**
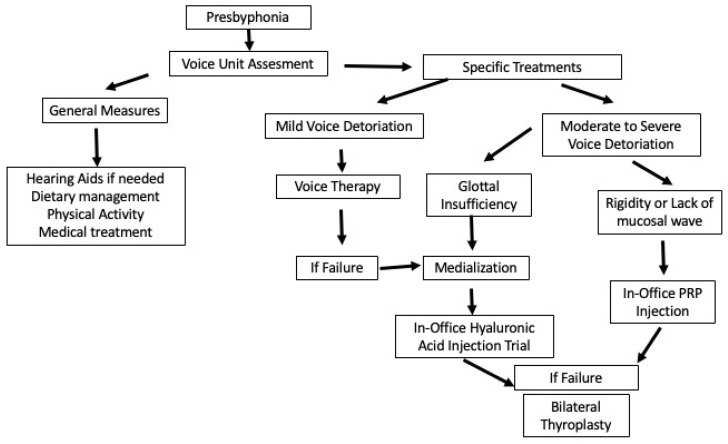
Algorithm proposed for management of presbyphonia.

A common approach is to initially opt for non-permanent solutions, such as office-based hyaluronic acid injections, reserving thyroplasty for cases where further intervention is needed. However, the effectiveness of medialization in presbyphonia is limited compared to unilateral vocal fold paralysis due to the persistent stiffness of the vocal folds [34]. To address this limitation, regenerative treatments such as platelet-rich plasma (PRP) injections can be considered. As Haddad suggests, injection laryngoplasty with hyaluronic acid is typically the first-line treatment when glottal gaps exist without vocal rigidity, while PRP injections are more appropriate for cases with vocal rigidity or mucosal deficiencies (Figure 5).

This study has certain limitations, such as its retrospective design and small final sample size (*n* = 16). Therefore, if the number of patients was increased, the results would be more significant. In addition, it should be taken into account that voice perception is very subjective, so scales such as VHI-10 and GRBAS were used. Additionally, pre-operative GRBAS scores were not collected for these patients, which may pose a problem when comparing voice quality after the infiltration. In order to measure the closure defect, it is necessary to download the ImageJ program and know how to use it, which may present an additional challenge as well.

## 5. Conclusions

In most patients with presbyphonia infiltrated with HA, there is a clear improvement in the glottic closure defect and satisfactory subjective sensation. This demonstrates the usefulness of this treatment in patients over 65 years old as a step preceding more durable treatments.

## Figures and Tables

**Table 1:** Results of 16 patients infiltrated with hyaluronic acid. Abbreviations: M, male; F, female; VHI-10, Voice Handicap Index-10; GRBAS, grade, roughness, breathiness, asthenia, strain.

Cases	Sex	Age	VHI-10 Before	VHI-10 After	GRBAS After	Pre-op Defect	Postop Defect	Closure Defect Improvement
1	F	78	31	23	2	7947.19	0	7947.19 (100%)
2	M	71	36	10	3	14,133.39	11,888.39	2245 (16%)
3	F	79	-	-	4	55,853.29	0	55,853.29 (100%)
4	F	70	18	15	3	3882.73	0	3882.73 (100%)
5	M	85	34	30	10	50,484.44	2369.42	48,115.02 (95%)
6	M	73	24	20	4	17,870.63	4367.98	13,502.65 (75%)
7	F	67	17	5	9	23,883.68	10,056.28	13,827.4 (58%)
8	M	71	20	12	6	35,225.14	0	35,225.14 (100%)
9	F	76	36	32	6	14,155.28	0	14,155.28 (100%)
10	F	75	26	12	7	50,821.76	24,194.15	26,627.61 (52%)
11	F	65	24	21	7	32,486.06	0	32,486.06 (100%)
12	F	73	22	17	5	7240.57	0	7240.57 (100%)
13	F	73	36	33	7	20,891.26	14,851.48	6039.78 (29%)
14	F	85	19	13	3	19,239.56	0	19,239.56 (100%)
15	M	78	21	13	8	7130.87	0	7130.87 (100%)
16	F	66	26	36	5	8464.73	0	8464.73 (100%)

**Table 2:** Comparison of improvement in closure defect with treatment duration and postoperative VHI-10. Abbreviations: VHI-10, Voice Handicap Index-10.

	Improvement Closure Defect >80%	Improvement Closure Defect <80%
**Duration > 9 months**	6	2
**Duration < 9 months**	5	3
**VHI-10 > 19**	5	2
**VHI-10 < 19**	5	3

## Data Availability

Data is contained within the article.

## Supplementary Materials

The following supporting information can be downloaded at: https://www.mdpi.com/article/10.3390/jcm14030960/s1. Video S1: Trans-cricothyroid HA infiltration. Video S2: Trans-thyrohyoid HA infiltration. Video S3: Defect area measurement using the ImageJ program.
